# Survival of patients with transformed follicular lymphoma in the United States: a multiple cohort study

**DOI:** 10.1186/s40364-023-00525-1

**Published:** 2023-09-27

**Authors:** John L. Vaughn, Narendranath Epperla

**Affiliations:** 1 Northeast Medical Group, Yale New Haven Health, 20 York St, CB 2041, New Haven, CT 06510 USA; 2 Department of Medicine, Division of Hematology, The Ohio State University, Columbus, OH USA

**Keywords:** Diffuse large B-cell lymphoma, Epidemiology, Follicular lymphoma, Transformed follicular lymphoma, SEER program

## Abstract

Population-based data comparing the outcomes of patients with transformed follicular lymphoma (t-FL) and de novo diffuse large B-cell lymphoma (DLBCL) are lacking. The objective of this study was to compare the survival of patients with t-FL and de novo DLBCL diagnosed in the United States between 2010–2018. We hypothesized that patients with t-FL would have an inferior survival compared to patients with de novo DLBCL. The study outcomes were relative survival (RS), overall survival (OS), and lymphoma-specific survival (LSS) compared between t-FL and de novo DLBCL. Flexible parametric survival models were used to estimate the study outcomes. There were 569 cases of t-FL and 44,706 cases of de novo DLBCL. Patients with t-FL had an estimated 5-year RS of 54% [95% confidence interval (CI), 49–59%) compared to 67% (95% CI, 66–67%) for those with de novo DLBCL (adjusted hazard ratio, 1.29; 95% CI, 1.11–1.50; *P* = 0.001). The corresponding 5-year OS estimates were 49% (95% CI, 44–53%) and 57% (95% CI, 57–58%), respectively (adjusted hazard ratio, 1.23; 95% CI, 1.07–1.42; *P* = 0.004). The corresponding 5-year LSS estimates were 54% (95% CI, 50–59%) and 66% (95% CI, 65–66%), respectively (adjusted hazard ratio, 1.34; 95% CI, 1.15–1.56; *P* < 0.001). This population-based registry analysis shows that patients with t-FL continue to have an inferior survival in the modern era and should be prioritized for enrollment in clinical trials.

**Supplementary Information:**

The online version contains supplementary material available at 10.1186/s40364-023-00525-1.

## To the Editor,

The treatment of B-cell lymphomas has advanced considerably over the past decade. However, only a few studies have reported the survival of transformed follicular lymphoma (t-FL) during this time period. Hence, we sought to compare the outcomes of t-FL and de novo diffuse large B-cell lymphoma (DLBCL) in the US population. We hypothesized that patients with t-FL would have an inferior survival compared to patients with de novo DLBCL.

We conducted a population-based, multiple cohort study of patients with t-FL and de novo DLBCL in the US using the Surveillance, Epidemiology, and End Results (SEER)-18 database. The SEER-18 database covers approximately 27.8% of the U.S. population based on the 2010 US Census. We included adults with FL who met the following inclusion criteria: (1) age 18–84 years at the time of diagnosis of FL, (2) diagnosed with FL between 2010–2014, (3) microscopic confirmation of their disease, and (4) FL was their first malignant primary tumor. The upper age limit was restricted to 84 years of age to allow for adequate follow-up time. We excluded individuals who met the following exclusion criteria: (1) diagnosed by autopsy or death certificate and (2) primary central nervous system (CNS) lymphomas. We identified patients with t-FL by following patients from their diagnosis of FL to their subsequent diagnosis of DLBCL through the end of 2018. All patients with t-FL had biopsy-proven rather than clinically suspected transformed disease.

We included adults with de novo DLBCL who met the following inclusion criteria: (1) age 18–99 years at the time of diagnosis of DLBCL (2) diagnosed with DLBCL between 2010–2018, (3) microscopic confirmation of their disease, and (4) DLBCL was their first malignant primary tumor. We excluded individuals who met the following exclusion criteria: (1) diagnosed by autopsy or death certificate and (2) primary CNS lymphomas. FL and DLBCL were identified using the SEER Lymphoid Neoplasm Recode 2021 Revision variable. Primary CNS lymphomas were identified by using the International Classification of Disease for Oncology topology codes C700-701, C709-729, and C751-753. Patients with composite DLBCL and FL (i.e., DLBCL and FL present in the same biopsy at initial diagnosis) were grouped with the de novo DLBCL patients by SEER.

The study outcomes were relative survival (RS), overall survival (OS), and lymphoma-specific survival (LSS). RS was defined as the ratio of all-cause to expected survival following diagnosis of lymphoma. Expected survival was estimated by matching patients with lymphoma to those in the general population by age, sex, year, and race using data from SEER. OS was defined as the probability of death from any cause following diagnosis of lymphoma. LSS was defined as the probability of survival when lymphoma was considered the only possible cause of death. Covariates were age at diagnosis, year of diagnosis, sex, race (White, Black, and Other), Ann Arbor stage (stage I-II and stage III-IV), B symptoms, and geographic region (Northeast, Midwest, South, and West). Age and year were modelled as continuous variables using restricted cubic splines with 3 knots. Age was also treated as a binary variable for specific analyses (< 65 years and ≥ 65 years).

Patient characteristics were analyzed using descriptive statistics. Differences between categorical and continuous variables were tested using Pearson’s chi-squared test and the Wilcoxon rank-sum test, respectively. Median follow-up time was estimated using the reverse Kaplan–Meier method [1]. The study outcomes were estimated using flexible parametric survival models with 6 knots for the baseline cumulative hazard [2]. Multivariable modeling was used to compare outcomes between patients with t-FL and de novo DLBCL by using histologic transformation as the key independent variable and adjusting for the study covariates. Histologic transformation was modelled as a time-dependent variable using restricted cubic splines with 3 knots. Missing data were handled using multiple imputation with chained equations. [3] *P*-values less than 0.05 were considered significant. Analyses were performed using Stata/IC version 16.1 (College Station, TX).

**Fig. 1:**
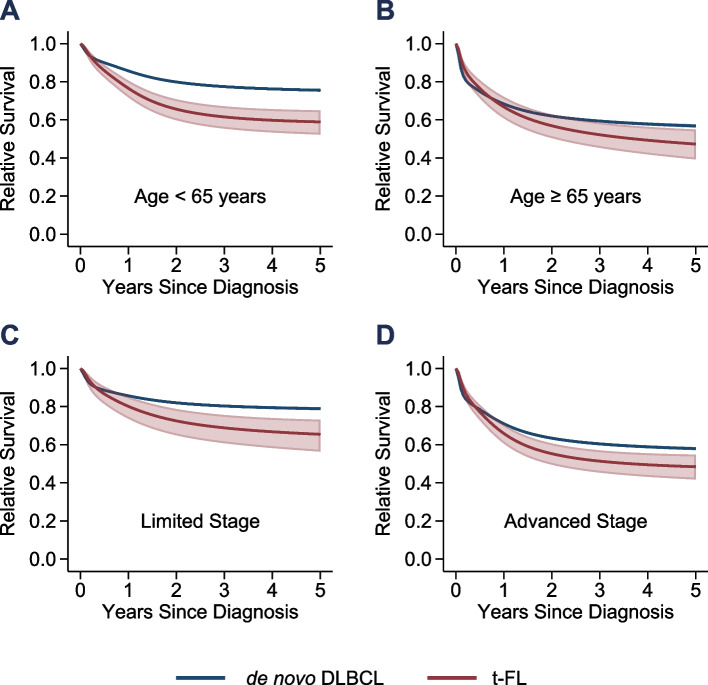
Relative survival of patients with de novo DLBCL and t-FL by age and stage. **A** Age < 65 years. **B** Age ≥ 65 years. **C** Limited stage. **D** Advanced stage. The start of each survival curve corresponds to the date of diagnosis of DLBCL, either transformed or de novo. 95% confidence intervals are shown but are not visible for de novo DLBCL due to the larger sample size

Among the 12,038 patients with FL who were included in the study, 569 (4.7%) patients developed biopsy-confirmed histologic transformation (HT) at a median follow-up of 6.3 years. A total of 44,706 patients with de novo DLBCL were also included in the study. Table S1 shows the key patient characteristics for t-FL and de novo DLBCL. Patients with t-FL had an estimated 5-year RS of 54% (95% CI, 49–59%) from their diagnosis of HT compared to 67% (95% CI, 66–67%) for de novo DLBCL (adjusted hazard ratio, 1.29; 95% CI, 1.11–1.50; *P* = 0.001). The corresponding 5-year LSS estimates were 54% (95% CI, 50–59%) and 66% (95% CI, 65–66%), respectively (adjusted hazard ratio, 1.34; 95% CI, 1.15–1.56; *P* < 0.001). The corresponding 5-year OS estimates were 49% (95% CI, 44–53%) and 57% (95% CI, 57–58%), respectively (adjusted hazard ratio, 1.23; 95% CI, 1.07–1.42; *P* = 0.004). The estimated median OS for patients with t-FL was 4.6 years (95% CI, 2.9–6.3) compared to 8.8 years (95% CI, 8.4–9.3) for those with de novo DLBCL. Table S2 shows the results of the multivariable modeling for each study outcome. Figure 1 shows the difference in RS based on age and stage at diagnosis.

We found that patients with t-FL had significantly lower survival compared to those with de novo DLBCL diagnosed between 2010–2018. This difference in survival was significant across study outcomes. Our results show that patients with t-FL were more likely to die from lymphoma compared to de novo DLBCL, which was supported using both cause-specific and relative survival frameworks. The differences in survival were larger for younger patients and those with limited stage disease. These findings stand in contrast to those from smaller cohort studies that did not observe a difference in survival between patients with t-FL and de novo DLBCL [4–7]. Possible explanations for our findings include differences in the disease biology (t-FL versus de novo DLBCL) and receipt of treatment regimens. Patients with t-FL may have more aggressive disease biology or may be less likely to receive anthracycline-based chemoimmunotherapy regimens if they already received them for follicular lymphoma. An additional factor to consider is the exclusion of these t-FL patients from the majority of the clinical trials that are primarily intended for FL or de novo DLBCL.

The cumulative incidence of biopsy-proven HT in our study was 4.7% (569/12,038). This was lower than the cumulative incidence of 5.5% (147/2652) reported by the National LymphoCare study [8]. The patients in that study were enrolled between 2004–2007 compared to 2010–2014 in our study. The lower cumulative incidence in our study may be due to differences in biopsy practices across locations and time, a declining rate of HT in the US population, or shorter follow-up duration (6.3 years in our study vs 6.8 years in the National LymphoCare study). Although we did not include patients with clinically suspected HT, these patients were shown to have the same survival as those with biopsy-proven disease [9].

Our study is limited by the lack of granularity pertaining to the treatment and molecular data as well as information on variables necessary to calculate International Prognostic Index scores. As we did not have access to the month of diagnosis for each patient, we were also unable to estimate the exact time-to-transformation. Lastly, it could be possible that a small number of patients with composite FL and DLBCL were included in the *de* novo DLBCL group; however, the number of patients with composite disease would not be large enough to alter the main findings of this study.

In conclusion, this is the largest study ever reported for t-FL. This is also the first population-based study to compare the outcomes of t-FL and de novo DLBCL*.* Patients with t-FL should be prioritized for clinical trials given the relatively inferior prognosis of these patients. Future observational studies should incorporate treatment information and molecular data to better understand the underlying mechanism driving the differential outcome of t-FL and de novo DLBCL observed in our study.

### Supplementary Information

**Additional file 1: Supplementary Appendix. Table S1.** Characteristics of adults diagnosed with *de novo *DLBCL and t-FL in the United States, SEER-18 database, 2010-2018. **Table S2.** Flexible parametric survival models for adults diagnosed with t-FL and *de novo *DLBCL in the United States, SEER-18 database, 2010-2018

## Data Availability

Not applicable.
