# Supplementary material for: Survival of patients with transformed follicular lymphoma in the United States: a multiple cohort study
Source: Biomark Res. 2023 Sep 27;11:84. doi: 10.1186/s40364-023-00525-1 (PMC10523601; doi:10.1186/s40364-023-00525-1)
Supplement: Supplementary file 1 — Additional file 1: Supplementary Appendix. Table S1. Characteristics of adults diagnosed with de novo DLBCL and t-FL in the United States, SEER-18 database, 2010-2018. Table S2. Flexible parametric survival models for adults diagnosed with t-FL and de novo DLBCL in the United States, SEER-18 database, 2010-2018 [file 40364_2023_525_MOESM1_ESM.docx]

**Supplementary Appendix**

**Table S1.** Characteristics of adults diagnosed with *de novo* DLBCL and t-FL in the United States, SEER-18 database, 2010-2018

**Table S2.** Flexible parametric survival models for adults diagnosed with t-FL and *de novo* DLBCL in the United States, SEER-18 database, 2010-2018

**Table S1.** Characteristics of adults diagnosed with *de novo* DLBCL and t-FL in the United States, SEER-18 database, 2010-2018

|  | ***de novo* DLBCL** | **t-FL** | **p-value** |
| --- | --- | --- | --- |
|  | N=44,706 | N=569 |  |
| Median age at diagnosis, y (IQR) | 66 (55-76) | 64 (56-72) | 0.01 |
| Age group |  |  | <0.001 |
| 18-44 | 5,405 (12%) | 20 (4%) |  |
| 45-54 | 5,712 (13%) | 101 (18%) |  |
| 55-64 | 9,616 (22%) | 171 (30%) |  |
| 65-74 | 10,875 (24%) | 171 (30%) |  |
| 75-99 | 13,098 (29%) | 106 (19%) |  |
| Sex |  |  | 0.65 |
| Female | 19,849 (44%) | 258 (45%) |  |
| Male | 24,857 (56%) | 311 (55%) |  |
| Race |  |  | <0.001 |
| White | 36,320 (81%) | 511 (90%) |  |
| Black | 3,545 (8%) | 22 (4%) |  |
| Other | 4,426 (10%) | 34 (6%) |  |
| Missing | 415 (1%) | 2 (0%) |  |
| Year of diagnosis |  |  | <0.001 |
| 2010 | 4,660 (10%) | 9 (2%) |  |
| 2011 | 4,722 (11%) | 32 (6%) |  |
| 2012 | 4,887 (11%) | 57 (10%) |  |
| 2013 | 4,872 (11%) | 81 (14%) |  |
| 2014 | 5,019 (11%) | 114 (20%) |  |
| 2015 | 5,075 (11%) | 79 (14%) |  |
| 2016 | 5,068 (11%) | 81 (14%) |  |
| 2017 | 5,194 (12%) | 51 (9%) |  |
| 2018 | 5,209 (12%) | 65 (11%) |  |
| Geographic Region |  |  | <0.001 |
| Northeast | 6,545 (15%) | 54 (9%) |  |
| Midwest | 4,225 (9%) | 78 (14%) |  |
| South | 9,318 (21%) | 103 (18%) |  |
| West | 24,618 (55%) | 334 (59%) |  |
| Ann Arbor stage |  |  | <0.001 |
| Stage I-II | 18,421 (41%) | 185 (33%) |  |
| Stage III-IV | 21,704 (49%) | 327 (57%) |  |
| Missing | 4,581 (10%) | 57 (10%) |  |
| B symptoms |  |  | <0.001 |
| No B symptoms | 24,500 (55%) | 328 (58%) |  |
| B symptoms | 12,657 (28%) | 95 (17%) |  |
| Missing | 7,549 (17%) | 146 (26%) |  |

Abbreviations: DLBCL = diffuse large B-cell lymphoma, t-FL = transformed follicular lymphoma, IQR = interquartile range

**Table S2.** Flexible parametric survival models for adults diagnosed with t-FL and *de novo* DLBCL in the United States, SEER-18 database, 2010-2018

|  | **RS Models** | | **OS Models** | | **LSS Models** | |
| --- | --- | --- | --- | --- | --- | --- |
|  | **HR (95% CI)** | **p-value** | **HR (95% CI)** | **p-value** | **HR (95% CI)** | **p-value** |
| Unadjusted |  |  |  |  |  |  |
| *de novo* DLBCL | Reference |  | Reference |  | Reference |  |
| t-FL | 1.34 (1.16-1.54) | <0.001 | 1.21 (1.07-1.37) | 0.002 | 1.37 (1.20-1.56) | <0.001 |
| Adjusted* |  |  |  |  |  |  |
| *de novo* DLBCL | Reference |  | Reference |  | Reference |  |
| t-FL | 1.29 (1.11-1.50) | 0.001 | 1.23 (1.07-1.42) | 0.004 | 1.34 (1.15-1.56) | <0.001 |

*Adjusted for age, year, sex, race, Ann Arbor stage, B symptoms, and geographic region.

Abbreviations: CI = confidence interval, DLBCL = diffuse large B-cell lymphoma, EHR = excess hazard ratio, HR = hazard ratio, LSS = lymphoma-specific survival, OS = overall survival, RS = relative survival, t-FL = transformed follicular lymphoma
